# Microbial Functional Diversity, Biomass and Activity as Affected by Soil Surface Mulching in a Semiarid Farmland

**DOI:** 10.1371/journal.pone.0159144

**Published:** 2016-07-14

**Authors:** Yufang Shen, Yingying Chen, Shiqing Li

**Affiliations:** 1 State Key Laboratory of Soil Erosion and Dryland Farming on Loess Plateau, Northwest A&F University, Yangling 712100, Shaanxi, China; 2 Institute of Soil and Water Conservation, Chinese Academy of Sciences and Ministry of Water Resources, Yangling 712100, Shaanxi, China; Old Dominion Univ., UNITED STATES

## Abstract

Mulching is widely used to increase crop yield in semiarid regions in northwestern China, but little is known about the effect of different mulching systems on the microbial properties of the soil, which play an important role in agroecosystemic functioning and nutrient cycling. Based on a 4-year spring maize (*Zea mays* L.) field experiment at Changwu Agricultural and Ecological Experimental Station, Shaanxi, we evaluated the responses of soil microbial activity and crop to various management systems. The treatments were NMC (no mulching with inorganic N fertilizer), GMC (gravel mulching with inorganic N fertilizer), FMC (plastic-film mulching with inorganic N fertilizer) and FMO (plastic-film mulching with inorganic N fertilizer and organic manure addition). The results showed that the FMO soil had the highest contents of microbial biomass carbon and nitrogen, dehydrogenase activity, microbial activity and Shannon diversity index. The relative use of carbohydrates and amino acids by microbes was highest in the FMO soil, whereas the relative use of polymers, phenolic compounds and amines was highest in the soil in the NMC soil. Compared with the NMC, an increased but no significant trend of biomass production and nitrogen accumulation was observed under the GMC treatment. The FMC and FMO led a greater increase in biomass production than GMC and NMC. Compare with the NMC treatment, FMC increased grain yield, maize biomass and nitrogen accumulation by 62.2, 62.9 and 86.2%, but no significant difference was found between the FMO and FMC treatments. Some soil biological properties, i.e. microbial biomass carbon, microbial biomass nitrogen, being sensitive to the mulching and organic fertilizer, were significant correlated with yield and nitrogen availability. Film mulching over gravel mulching can serve as an effective measure for crop production and nutrient cycling, and plus organic fertilization additions may thus have improvements in the biological quality of the soil and its sustainability in the rainfall-limited semiarid region.

## Introduction

Drylands account for approximately 55% of the area of China’s cultivated land and are indispensable for meeting the food demand of an ever-increasing population. Mulching has been widely used to increase agricultural productivity of dry farmland, especially in the arid and semiarid areas of China where crop production is constrained by limited precipitation, high evaporation and nutrient-poor soil [1, 2]. Gravel with its availability and low cost has been a preferred mulch for many years [3], and plastic-film mulching has become a well-evolved technique in agriculture since the 1990s in the semiarid areas of northwestern China. These mulching technologies are highly effective in increasing soil moisture and topsoil temperature [4, 5], improving nutrient availability [6] and thus significantly improving yield and water-use efficiency [3, 7]. Microbial biomass, activities and diversities respond quickly to the changes in soil conditions, and the changes can be valuable indicators of the effect of management on the soil environment and can have large impacts on ecosystemic dynamics. For example, Kong et al. [8] suggested that fungal activity was a potential indicator of the quality of soil microenvironments, having an important role in carbon processing. Gomez et al. [9] suggested that the microbial functional diversity by carbon source use profiles was also sensitive to changes in the short term due to management practices. The organic amendment soil had significant increases in AWCD, richness and Shannon-Weaver index, and linear relationship between the microbial functional diversity and soil carbon availability [9]. However, in some case, the microbial populations of rice field soil subjected to organic and conventional farming presented similar functional and bacterial richness and diversity and enzymatic activity [10]. Soil microorganisms constitute a source and sink for nutrients and variations in the structure of microbial communities can also influence the rates of nitrification, de-nitrification and nitrogen fixation [11], which is related to nutrient cycles and thus crop production. Whether the increased grain yield and nutrient availability were consequences of improved microbial metabolic capabilities, however, is not known. A better understanding of the effects of long-term mulching on microbial activity and diversity would thus be conducive to maintaining soil fertility and productivity by enabling the design of the best farming strategies. Concerns about the sustainability of the agricultural ecosystem have also increased the interest in microbial communities and how they and their functions are affected by special environmental factors, such as changed soil pH, the availability of labile C and N and soil water.

Crop production is a comprehensive exercise, and yield varies widely in response to a variety of changes. Fertilizers, especially nitrogen (N) fertilizers, have played an important role in increasing crop production since the 1950s [12]. The use of costly chemical fertilizers, however, can be minimized or replaced by the use of locally available manure [13–15]. The application of manure or compost can improve soil organic-carbon content and may also enhance soil microbial activities. Microorganisms are involved in numerous processes, such as transformation of C and N, formation of soil physical structure [14, 16], which is important for improving soil properties to sustain crop productivity and environmental quality [15]. Manure, though, can have positive or negative effects on microbial diversity, biomass and activity in agricultural ecosystems [17–18]. Van Groenigen et al. [19] reported that the flow of carbon (C) through ecosystems from agricultural management was largely mediated by soil microorganisms, and microbial communities have the ability to respond rapidly to changing environmental conditions by modifying biomass and community composition [20]. Any changes in the type or amount of organic matter entering the soil can thus directly affect soil enzymatic activity, microbial biomass and activity, microbial community or functions performed by the various microbial groups [21, 22].

Monocultured spring maize (*Zea mays* L.) has been widely adopted on the Loess Plateau, a typical semiarid region in northwestern China [23]. Most studies have concentrated on the effect of plastic-film mulching and fertilizers on plants and on soil temperature, water content and nutrient content [1, 24], but the influence of the practices of agricultural management on the microbial properties of the maize rhizosphere remains poorly understood. Understanding how the microbial properties respond to the practices is critical to the maintenance of soil quality and is essential for sustaining intensive agricultural production for meeting the food demands of growing populations. The objectives of this study were to (1) assess the impact of different mulching patterns and organic manure addition at film mulching on soil microbial biomass, soil enzyme activity and microbial functional diversity in the current sustaining intensive maize production system, and (2) identify the changes in soil microbes contributing to yield improvement and nutrient availability. The results will provide essential knowledge for determining the best practices of agricultural management for sustaining crop production in this semiarid farmland.

## Materials and Methods

### Ethics statement

The study was conducted at the Changwu Agricultural Experimental Station of Northwest A&F University on the Loess Plateau of China. Permission was obtained from the Station administration to allow conducting the field experiment. Further, the experimental station where field study conducted was not protected location for endangered or protected species.

### Site description and experimental design

**Fig 1:**
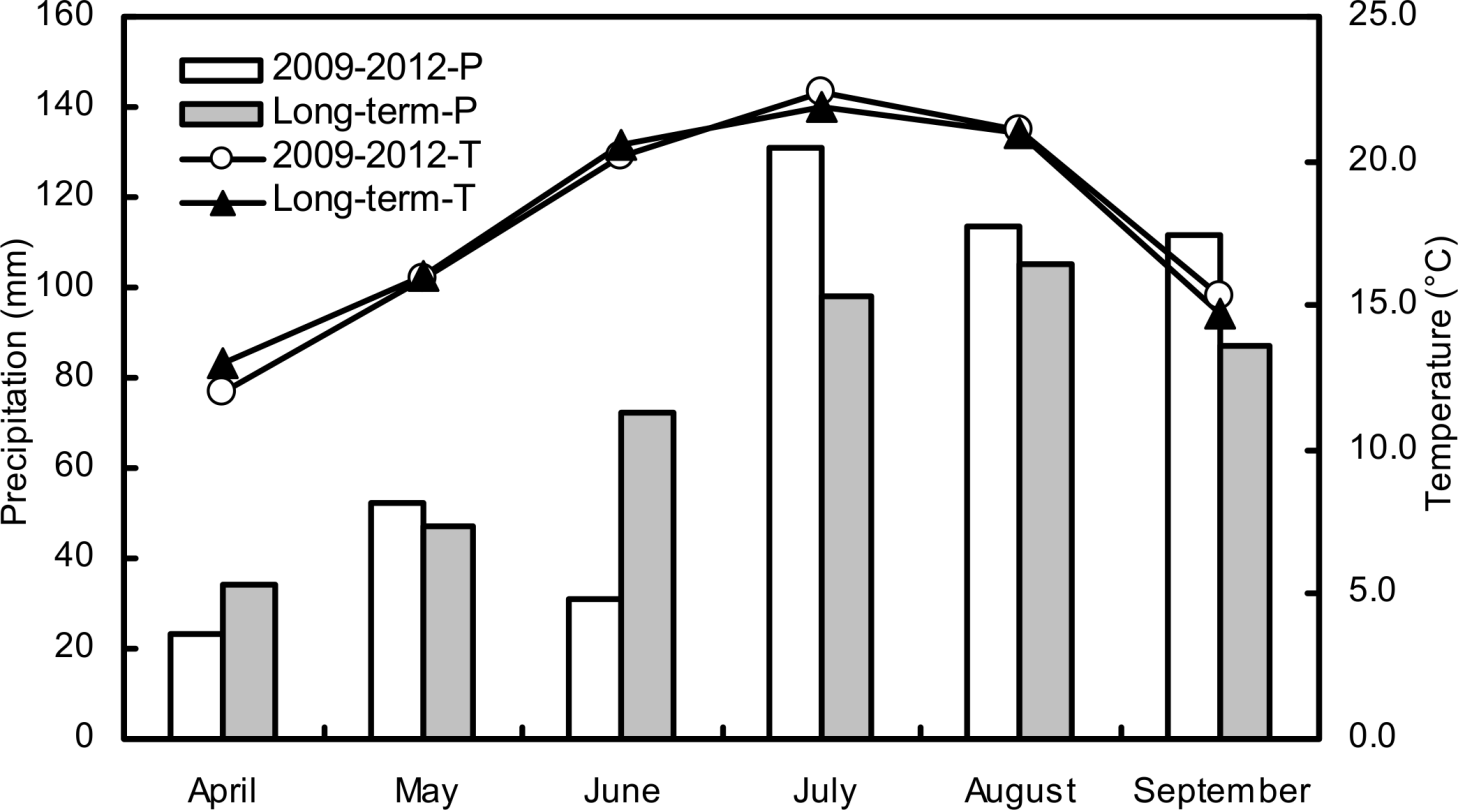
The mean monthly precipitation (P) and air temperature (T) during the maize growing season in the five experimental years and long term (last 20 years) at the study site.

Untilled triplicate experimental plots (8 × 7 m) were established in a completely randomized split-plot design in April 2009 at the Changwu Agricultural Experimental Station on the Loess Plateau of China (35.28°N, 107.88°E, approximately 1200 m above sea level). Dryland farming is dominated by monocultured cropping systems that are mainly comprised of spring maize, which is produced by rain-fed agriculture across the region. The average annual precipitation is 578 mm, of which about 73% falls during the spring maize growing season (May-September). The annual average temperature was 9.3°C, and the mean temperature between May and September was 19.0°C during the last 50 years. The temperature and mean daily precipitation at the site during the five years of the experiment were measured at the Changwu meteorological monitoring station (Fig 1). The field site has an average annual evaporation from a free water surface of 1565 mm, and groundwater, with water table at a depth >60 m, is unavailable for plant growth. The experimental soil had a silty loamy texture according to the USDA texture classification system. The soil at the beginning of the experiment in 2009 had a pH of 8.4 and contained 6.39 g kg^-1^, 0.87 g kg^-1^, 14.4 mg kg^-1^, 133.1 mg kg^-1^ and 28.8 mg kg^-1^ of organic C, total N, Olsen-phosphorus, NH_4_OAc-extractable potassium and mineralized N, respectively, and had a bulk density to a depth of 20 cm of 1.30 g cm^-3^.

We tested four treatments: (1) no mulching with inorganic N fertilizer (NMC), (2) gravel (2–4 cm in size) mulching with inorganic N fertilizer (GMC), (3) plastic-film mulching with inorganic N fertilizer (FMC) and (4) plastic-film mulching with inorganic N fertilizer and organic manure addition (FMO). The annual application rates for fertilzers were 225 kg N ha-1, 40 kg P ha-1 and 180 kg K ha-1. Forty percent of the N fertilizer was distributed over the soil surface prior to sowing and then was plowed into the subsurface as a basal dressing. The remaining two thirty percents of the N fertilizer was applied at the jointing stage and the silking stage. Manure (cow dung) was also applied seasonally at rate of 25 kg N ha^-1^prior to planting. All plots were planted with maize with alternating wide and narrow row spacings of 60 and 40 cm in the furrows separating adjacent ridges. The strips of plastic film in the FMC and FMO treatments were laid end-to-end along the longitudinal axis of the wider ridges.

The entire experimental area was plowed in 2009 before the plots were separated by earthen berms to prevent the movement of water between plots and from upslope of the plots. The plots were treated with a basal chemical nitrogen fertilizer and the manure after the ridges and furrows had been formed, and then plowed to incorporate the fertilizer into the subsurface. The maize in each plot was planted 5 cm deep with a hole-sowing tool in late April each year. The water supply for each treatment came solely from rain. Uniform weeding protection and pesticides were applied when necessary. The plots were harvested as the maize ripened in late September each year.

### Soil sampling

Soils were sampled from different treatments at the silking stage of maize in 2012. Soil cores were drilled randomly with three replications from each plot close to the selected plants using a soil corer to a depth of 20 cm at least 1 m from the plot edge and 0.5 m from previous sampling sites. The three sub-samples were then mixed into one composite sample for each plot. As described by Butler et al. [25], three maize plants with roots were also randomly collected from each plot and were shaken vigorously for about 1 min to dislodge the soil not tightly adhering to the roots. The remaining soil tightly attached to the roots was considered the rhizosphere. The rhizosphere soil was then carefully removed from the roots with a probe and forceps. The soil subsamples from the same plot were composited, placed in labeled plastic bags in the field and kept cool until transported to the laboratory where they were processed for subsequent analysis within 1 h after collection. All visible roots, fresh litter material, stones and coarse particles were removed manually, and the composite samples were passed through a 2-mm sieve, homogenized and stored at 4°C for the biochemical and microbial analyses. The samples were processed and analyzed within two weeks. The organic carbon of the collected soil was as follows: NMC 6.41 g kg^-1^, GMC 6.31 g kg^-1^, FMC 6.48 g kg^-1^ and FMO 6.55 g kg^-1^.

### Determination of microbial biomass and enzymatic activities

Soil microbial biomass C (MBC) and N (MBN) were measured by chloroform fumigation /extraction [26, 27]. The 0.5M K_2_SO_4_ extractable-C was analyzed by an automated total organic-C analyzer (TOC-Vcph, Shimadzu, Japan). MBC was calculated as the difference in extractable C between the fumigated and unfumigated soil using a *K*_c_ factor of 0.45 [28]. The 0.5M K_2_SO_4_ extractable-N was measured by dual-wavelength ultraviolet spectrophotometry. MBN was calculated as the difference in extractable N between the fumigated and unfumigated soils using a Kn factor of 0.54 [29].

Dehydrogenase activity is often used as a measure of microbial activity [30]. Dehydrogenase activity was determined according to a method described by Klose et al. [31]. Briefly, 2 g of moist rhizosphere soil were weighed in glass vials and treated with 2.5 ml of 1% triphenyltetrazolium chloride(TTC)-Tris buffer (pH 7.6). The suspensions were then incubated in the dark at 37°C for 24 h. After incubation, the triphenylformazan (TPF) produced was extracted with methanol and estimated colorimetrically. All measurements were carried out in triplicate with one blank. Results are expressed as mg of TPF released kg^-1^ soil d^-1^.

Urease activity is closely associated with soil nutrient content and can be used for estimating soil quality. Urease activity was determined using a modification of the method proposed by Kandeler and Gerber [32]. Two grams of air-dried soil were combined with 10 ml of 10% urea and 20 ml of citrate buffer (pH 6.7). The mixtures were then incubated for 24 h at 37°C. Controls were prepared without substrate to determine the production of ammonium ions in the absence of urea. The ammonium content was determined at 578 nm using a modified indophenol blue reaction.

### Community level physiological profiles (CLPP)

Biolog EcoPlates (Biolog Inc., Hayward, California, USA) were used to study the pattern of substrate use by the soil microbial communities based on the use of 31 C substrates described by Garland [33]. Color formation from a redox indicator dye was used to quantify the amount of a C source used. The rhizosphere soil samples were prepared and inoculated within two days of sampling. In brief, fresh soil (5 g) was added to 45 ml of sterile saline (0.85% NaCl, w/v) in a 250-ml flask and shaken for 30 min at 200 rpm to achieve a 10^−1^ dilution. Tenfold serial dilutions were prepared to 10^−3^. The wells of the Biolog EcoPlates were inoculated with 150 μl of the dilutions, and all plates were placed in polyethylene bags to reduce desiccation and incubated in dark growth chambers at 25°C for 240 h. Color development was measured every 12 h with an ELISA plate reader at a wavelength of 590 nm, and the data were collected using Microlog 4.01 (Biolog Inc.). Each treatment was replicated three times. The readings at 144 h were used for the statistical analysis. The microbial activity in each microplate well expressed as average well-color development (AWCD) was determined as: AWCD = Σ(Ci-R)/n (n = 31), where Ci is the optical density of substrate i measured at 590 nm and R is the optical density of the control.

The Shannon diversity index (*H*^*′*^) of the bacterial communities was calculated based on the data at 144 h of incubation:

$$H^{\prime }=-\sum_{i=1}^{n}\;Pi\;(\ln\;Pi)$$

where *Pi* is the ratio between the activity of each substrate and the sum of the activities of all substrates [34].

### Plant samples and aboveground nitrogen uptake

In each plot, the plant growth was monitored, and according to the standardized maize development stage system when 50% or more of the plants reached the silking stage, three adjacent plants in the same row were selected randomly and cut at ground level. At maturity, 10 m^2^ (four rows each 2.5 m long) area in the middle of each plot was manually harvested, and plant samples were divided into grain and straw to determine the crop biomass and grain yield. All sampled plants were dried initially at 105°C for 30 min and then dried to a constant weight at 80°C before being ground for the analysis of nitrogen content (micro-Kjeldahl). All masses are expressed as percentages of the dry weight. The N-use efficiency (NUE) was calculated as: NUE (kg kg^-1^) = GY/FN, where GY is the grain yield (kg ha^-1^) and FN is the application rate of the N fertilizer (kg ha^-1^).

### Statistical analysis

The means and standard deviations were calculated for all of the parameters. The differences between the treatment means were compared by using the least significant difference (LSD) and were deemed to be significant if P < 0.05. Principal component analyse (PCA) was used to identify distinction in the carbon source utilization patterns of soil microbial communities in different mulching patterns. The statistical analyses were performed using SPSS version16.0.

## Results

### Microbial biomass and enzymatic activities

**Fig 2:**
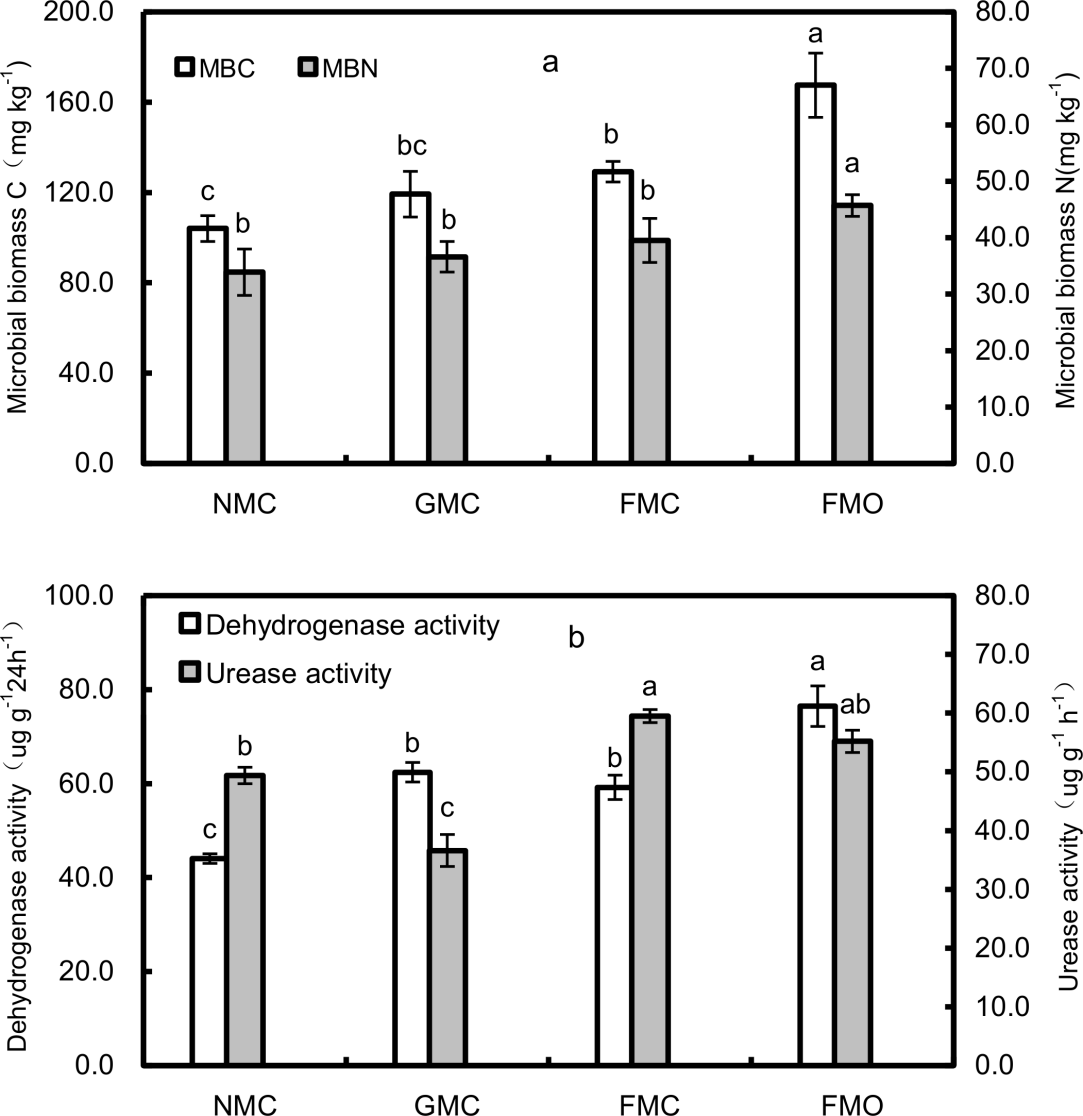
**The microbial biomass C and microbial biomass N (a) and dehydrogenase activity and urease activity (b) in each treatment. Soil samples were tested from rhizosphere of maize treated with different mulching and fertilizer treatments.** The values are the means of three replicates, and different letters above the bars indicate significant differences at the P < 0.05 level. NMC, no mulching with inorganic N fertilizers; GMC, gravel mulching with inorganic N fertilizers; FMC, plastic-film mulching with inorganic N fertilizers; FMO, plastic-film mulching with chemical N fertilizers and organic manure addition.

MBC, MBN and dehydrogenase and urease activities differed in the mulching and fertilizer treatments (Fig 2). The FMO soil had the highest MBC, MBN and dehydrogenase activity, and the FMC soil had the highest urease activity. MBC was 60.9% higher in the FMO than the NMC soil but did not differ significantly between the GMC and NMC soils (Fig 2A). MBN generally decreased in the order: FMO > FMC > GMC > NMC. The mulching treatments, however, did not have significant effects on MBN (Fig 2A), which ranged from a low of 33.9 mg kg^-1^ in NMC to a high of 39.5 mg kg^-1^ in FMC. The application of manure significantly increased dehydrogenase activity in the maize rhizospheres of the film-mulched treatments (Fig 2B). Dehydrogenase activity was significantly (*P*<0.05) higher in the FMO than the FMC soil and was significantly higher in the FMC and GMC than NMC soils. An ANOVA indicated that urease activity was significantly (*P*<0.05) lower in the GMC than the NMC soil but did not differ significantly between the NMC and FMO soils or between the FMO and FMC soils (Fig 2B).

### CLPP

**Fig 3:**
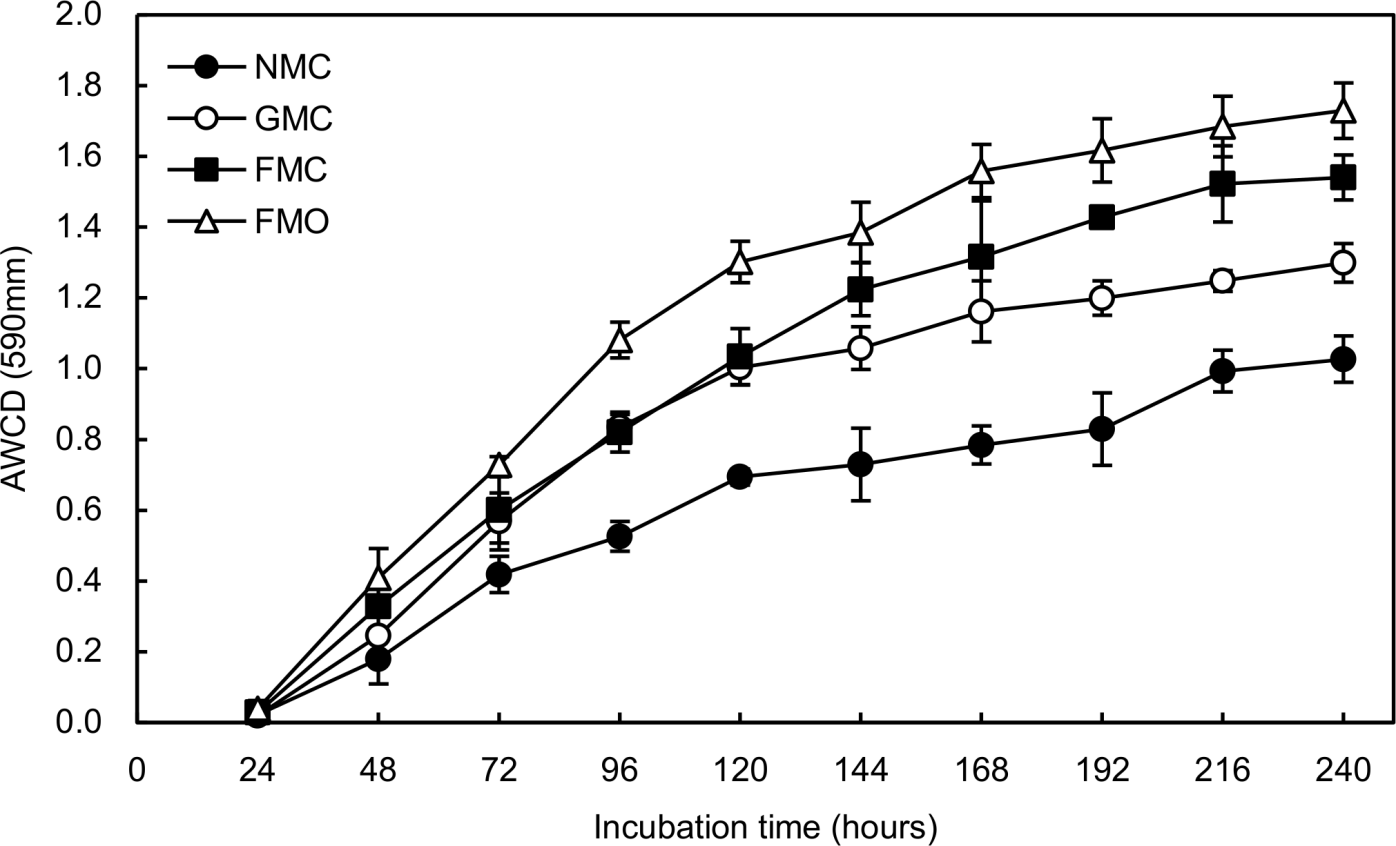
Variation in average well color development (AWCD) over time in Biolog ECO-microplates. Soil samples were tested from rhizosphere of maize treated with different mulching and fertilizer treatments. The values are the means of three replicates (means±SD).

AWCD is a direct indicator of the overall C-source metabolic activity of the soil microbes [35]. AWCD was nearly zero with no clear differences among all treatments before the first 24 h of incubation (Fig 3). AWCD gradually increased over time and differed significantly among all treatments at 48 h of incubation (*P*<0.05), although the AWCD of FMC and GMC was almost same at 96h incubation. The FMO soil had the highest AWCD, and the NMC soil had the lowest after 144h of incubation. AWCD for the NMC and GMC soil was 1.68–2.29-fold and 1.28–1.94-fold lower than for the FMO soils between 24 and 240 h of incubation, respectively.

**Fig 4:**
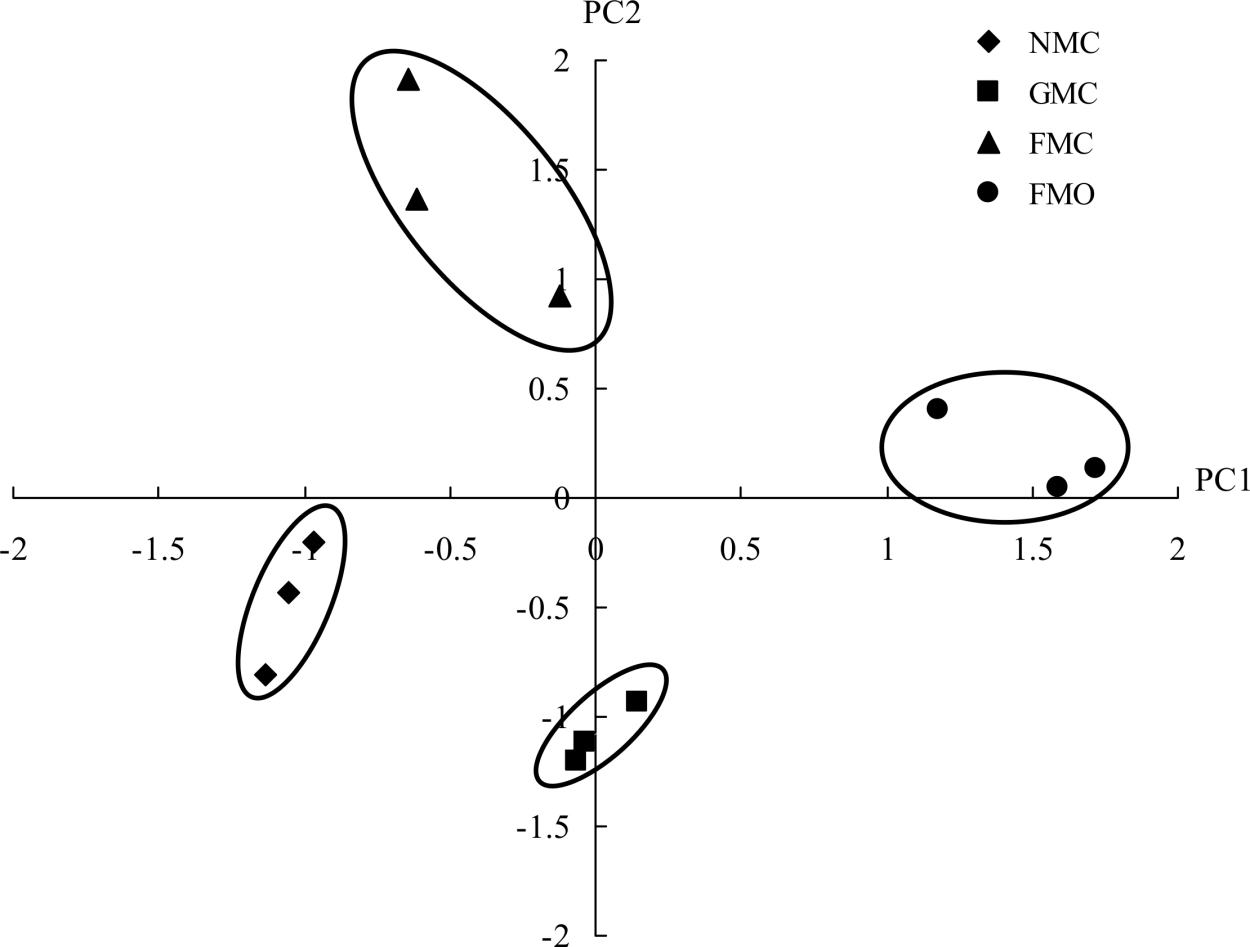
Principal component analysis of CLPP results from maize rhizosphere soil treated with different mulching and fertilizer treatments. Soil samples were tested from rhizosphere of maize treated with different mulching and fertilizer treatments.

**Fig 5:**
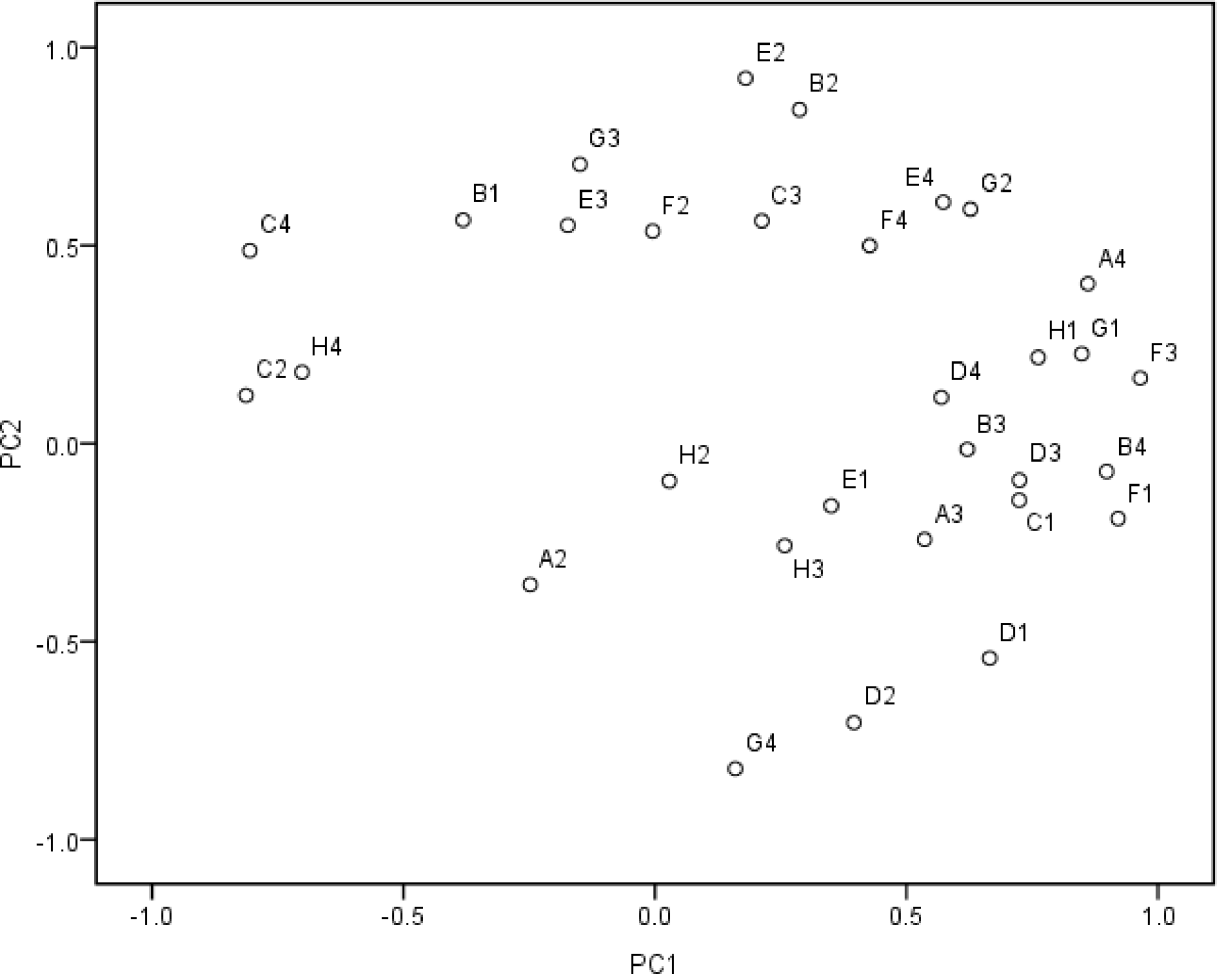
Loadings of 31 carbon substrates on PC1 and PC2 in the principal components analysis of the CLPP results. Carbon source: A1: Water, A2: β-methyl-D-glucoside, A3: D-galactonic acid γ-lactone, A4: L-arginine, B1: Pyruvic acid methyl ester, B2: D-xylose, B3: D-galacturonic acid, B4: L-asparagine, C1: Tween 40, C2: I-erythritol, C3: 2-hydroxybenzoic acid, C4: L-phenylalanine, D1: Tween 80, D2: D-mannitol, D3: 4-hydroxybenzoic acid, D4: L-serine, E1: α-cyclodextrin, E2: N-acetyl-D-glucosamine, E3: γ-hydroxybutyric acid, E4: L-threonine, F1: Glycogen, F2: D-glucosaminic acid, F3: Itaconic acid, F4: Glycyl-L-glutamic acid, G1: D-cellobiose, G2: Glucose-1-phosphate, G3: α-ketobutyric acid, G4: Phenylethylamine, H1:α-D-lactose, H2: D,L-α-glycerol phosphate, H3: D-malic acid, H4:Putrescine.

The PCA for the pattern of C-substrate use indicated that the first and second principal components explained 36.2 and 21.5% of the variance, respectively. The PCA suggested that the pattern of C-substrate use was significant affected by the mulching and fertilizer treatments (Fig 4). The treatments were divided into four groups based on the PCA scores. The FMO and FMC soils scored the highest on PC1 and PC2, respectively, and the NMC soils had negative scores on PC1 and PC2. The factor loadings of the 31 C sources on PC1 and PC2 are shown in Fig 5. F3 (Itaconic acid), F1 (Glycogen), B4 (L-asparagine), A4 (L-arginine), G1 (D-cellobiose), C2 (I-erythritol) and C4 (L-phenylalanine) made major contributions to PC1, whereas E2 (N-acetyl-D-glucosamine), B2 (D-xylose) and G4 (Phenylethylamine) made large contributions to PC2.

**Fig 6:**
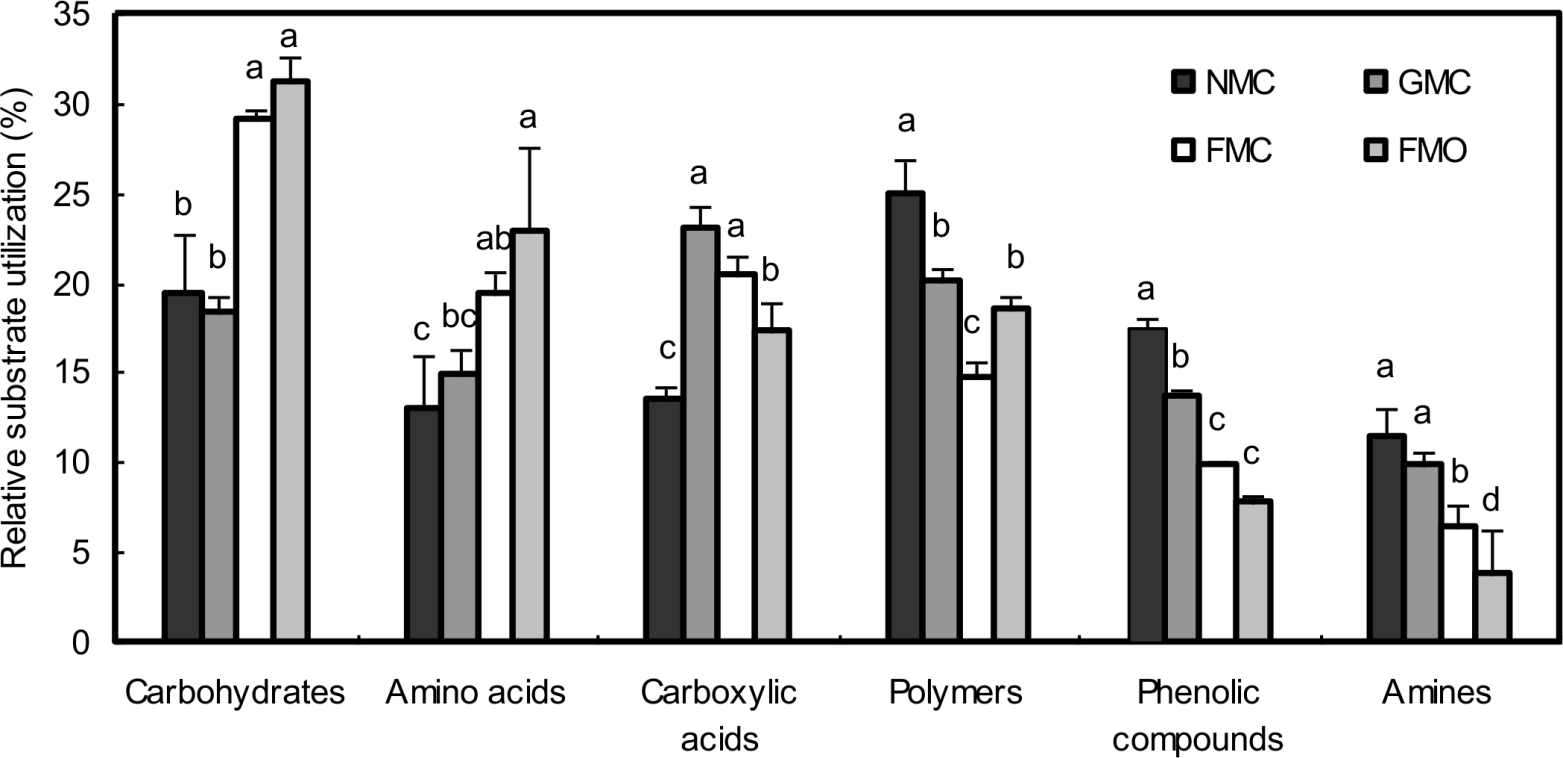
Relative substrate utilization for six substrate categories in each treatment. Soil samples were tested from rhizosphere of maize treated with different mulching and fertilizer treatments. The values are the means of three replicates, and different letters above the bars indicate significant differences at the P < 0.05 level.

The microbes could use all six substrate categories, and relative substrate utilization varying from 3.8 to 31.2%, but the pattern of C-substrate use was significantly affected by the mulching and fertilizer treatments (Fig 6). The relative use of carbohydrates and amino acids was higher in the FMO than the other soils and differed significantly from that in the GMC and NMC soils (*P*<0.05). The NMC soil used 24.9 and 17.6% polymers and phenolic compounds, which differed significantly from those for the GMC, FMC and FMO soils, respectively. The GMC soil had the highest relative use of carboxylic acids, which differed significantly from those for the NMC and FMO soils. Shannon diversity index (*H*^*′*^) at the 144h showed that the *H′* of FMO soil was 3.75, which was significantly higher than that for the NMC, GMC and FMC soils (*P*<0.05). Compared with the FMO soil, the *H′* under the NMC, GMC and FMC decreased by 16.3%, 17.6% and 21.9% respectively. *H′* did not differ significantly between the GMC and FMC soils.

### Biomass production, NUE and nitrogen accumulation

**Fig 7:**
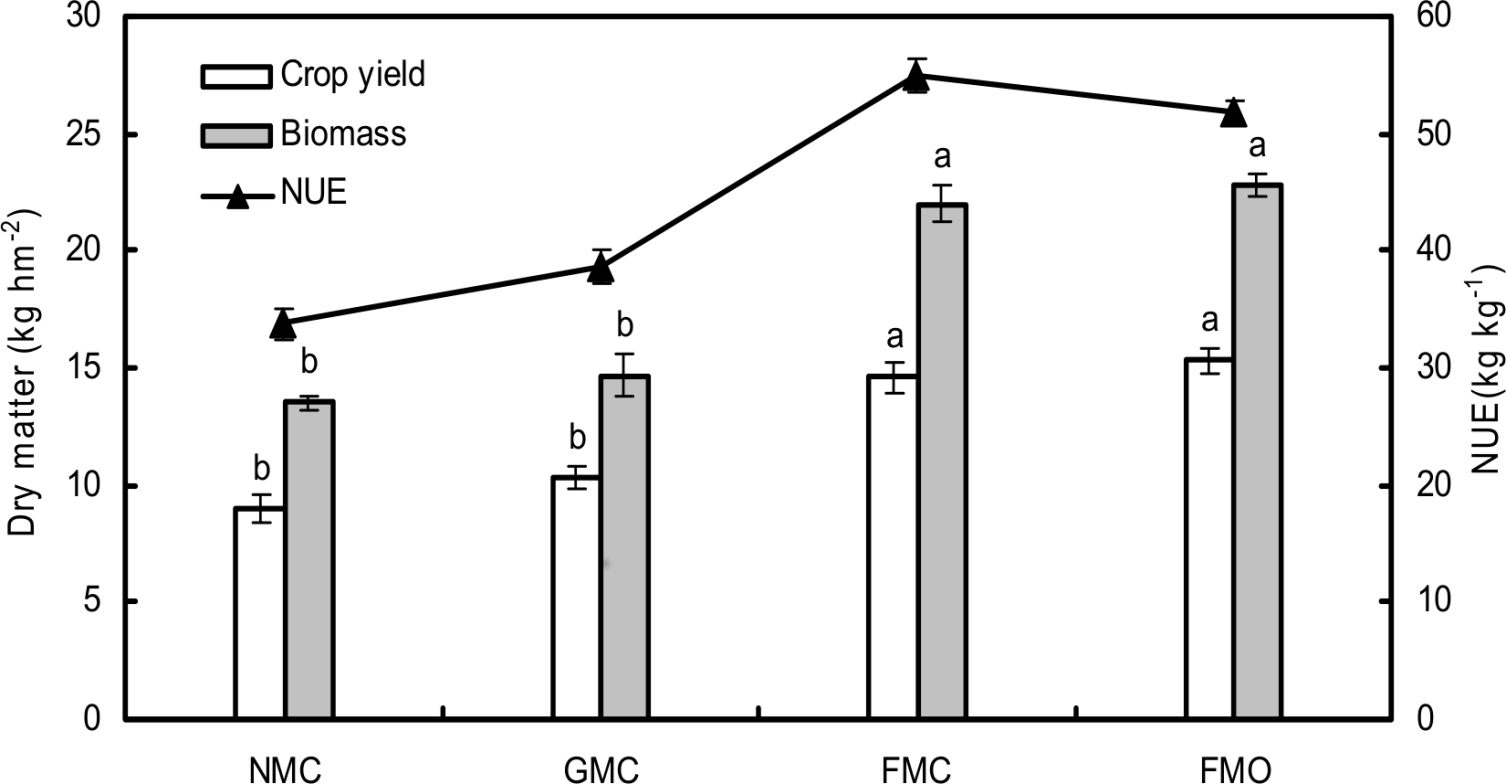
The grain yield, biomass and nitrogen use efficiency (NUE) of maize treated with different mulching and fertilizer treatments. The values are the means of three replicates, and different letters above the bars indicate significant differences at the P < 0.05 level.

Mulching stimulated maize biomass production and increased NUE (Fig 7). The GMC treatment increased the grain yield and maize biomass by 14.4 and 8.9%, respectively, compared to the NMC treatment, but the effects were not statistically significant. The FMC and FMO led a greater increase in biomass production than GMC and NMC.Compared with the NMC, the grain yield and maize biomass for the FMC treatments were significantly higher by 62.2 and 62.9%, and for the FMO treatments by 70.1 and 68.9%, respectively (*P*<0.05). No significant difference in grain yield or biomass was found between FMO and FMC treatment (Fig 7). Compared with the NMC treatment, NUE in the GMC, FMC and FMO treatments increased significantly. FMC had highest NUE, but did not differ significantly with FMO.

**Fig 8:**
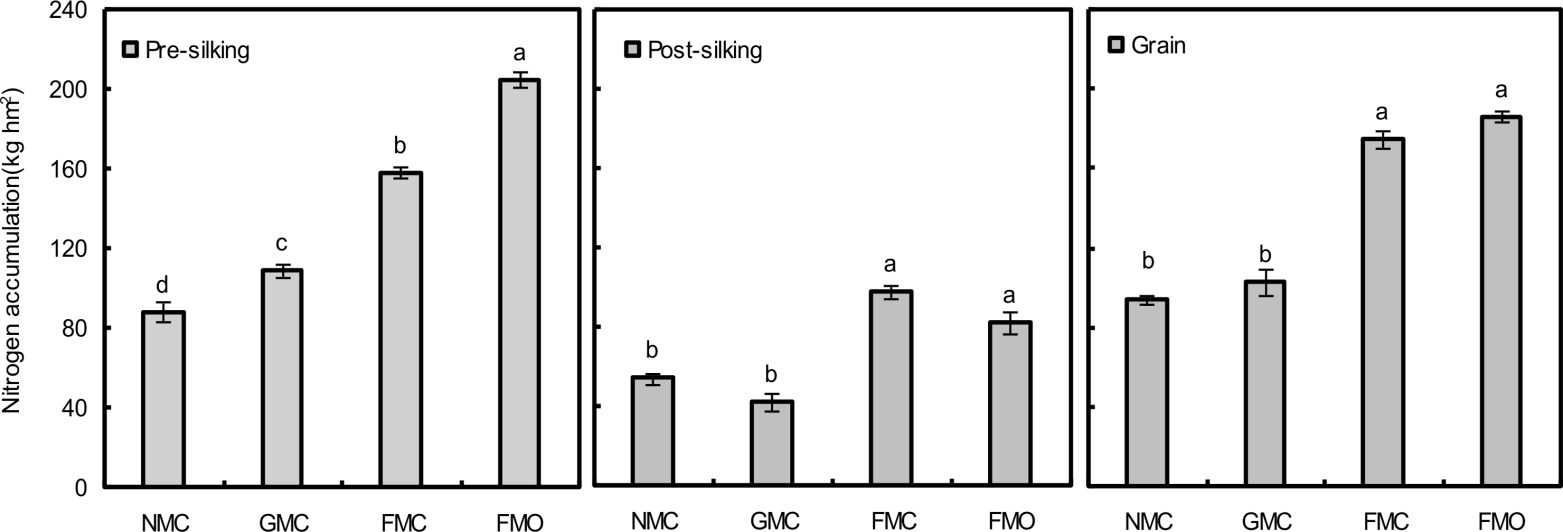
N accumulation during the pre- and post-silking period and of grain at maturity in different mulching and fertilizer treatments. The values are the means of three replicates, and different letters above the bars indicate significant differences at the P < 0.05 level.

Nitrogen accumulations during the pre- and post-silking stages and grain are shown in Fig 8. Nitrogen accumulation during the pre-silking stage represented 62–72% of the total aboveground nitrogen accumulation for all treatments. Mulching and fertilization had significant effects on nitrogen accumulation during the pre-silking stages (*P*<0.05). Compared with the NMC treatment, nitrogen accumulation in the GMC and FMC soil significantly increased (Fig 8). Compared with the FMC treatment, nitrogen accumulation in the FMO treatment increased by 29.7%. Film mulching had significant effect on nitrogen accumulation during the post-silking stage. Nitrogen accumulation was significantly higher in the FMC than the NMC soils, whereas no significant difference was found between the GMC and NMC treatments. Nitrogen accumulation in the FMO soil did not differ significantly from the FMC soils (Fig 8). Grain nitrogen accumulation tended to be higher at maturity in the order NMC < GMC < FMC < FMO (Fig 8). Compared with the NMC treatment, nitrogen accumulation in the GMC treatment increased by 9.6% (*P*>0.05), and in the FMO and FMC treatments increased by 97.9% and 86.2%, respectively (*P*<0.05). Grain nitrogen accumulation did not differ significantly between the FMO and FMC treatments (*P*>0.05).

## Discussion

### Microbial responses to mulching and fertilization

Practices of soil and crop management such as mulching and fertilization can have considerable effects on soil temperature, evaporation [3, 4], organic-matter content [16] and other measures of quality. Microorganisms, which drive most processes, respond quickly to these changes in conditions. Film or gravel-sand mulching modifies the physical and chemical environment of the soil and thus globally influences the growth and activity of microorganisms [36, 37], which in turn affects the physicochemical environment [38]. These changes play an important role in crop growth and agroecosystemic function and sustainability in dryland farming in northern China [24]. There was an increase trend in MBC in the FMC and GMC soil than NMC soil (Fig 2A), although the amplitude depended on the mulching method, which may mainly be attributed to the differences in soil temperature and moisture. Mulching can increase water flow to the roots of maize and minimize water losses by evaporation, leading to higher topsoil moisture content during the maize growing season [39]. The level of soil moisture has been associated with microbial biomass [40], but Zhang et al. [41] reported that plastic-film mulching reduced MBC and MBN during crop growth, which was in contrast to our results. The reason for the higher values in our study might attribute to the higher soil organic carbon content at the experimental site [38], since it had been suggested that its easily degradable part of the total DOC in soil resembled soil microbial activity [42]. In our study, mulching plus manure had higher microbial biomass and dehydrogenase activity compared with FMC treatment (Figs 2 and 3), consistent with previous studies [43, 44]. Gong et al. [43] reported that manure could provide abundant organic matter for the growth of microorganisms, with positive effects on microbial activity and biomass [44], and thus enzymatic activities [45]. Urease activity, however, did not clearly differ between the FMO and FMC soils after several years of continuous fertilizer application with and without manure, despite a decrease (Fig 2B). This result was in line with those of Jin et al. [46] that the specific activity of some enzymes did not necessarily reflect corresponding increases to the high soil organic carbon. Enzyme activities varied seasonally and, in most cases, some single enzyme was not representative of the overall microbial activity. Enzymatic activity may only control the availability of the most limiting nutrients for meeting microbial metabolic demands. Rolda´n et al. [45] reported that the increase of dehydrogenase activity together with the water-soluble C fraction after the adoption of no-tillage indicated water regime having no effect on total microbial activity in the maize field under subtropical conditions. Li et al. [47], however, reported that increased soil moisture or topsoil temperature under mulching technologies was closely related to soil microbial biomass. In this study, the increase of microbial activity could be possibly for the fact that improved soil water, with significant positive correlations were found between the soil water content and MBC and MBN (*R*^2^ = 0.546* and 0.378*). However, increased temperatures could also decrease the size of the soil microbial community after a longer time of soil warming, thus more studies of the relationship between soil microbial and soil moisture or temperature were obviously needed, for further understand the potential relationships between some enzymatic activities with the combined effects of favorable environmental factors in some agrosystems [48].

Microbial diversity, which has been associated with fertilization and management regimes [49, 50], is an important microbial parameter. Functional diversity, measured with the reliable Biolog method, is also widely used as a good indicator for evaluating and predicting changes of soil quality or productivity. In this study, the analyses performed using CLPP indicated that different mulching and organic fertilizer caused distinct changes of the potential microbial turnover in the soil, and lead to carbon utilisation pattern shifts. Zhong et al. [17] observed the higher AWCD and functional diversity indices by the addition of organic matter, and substrate utilization patterns were different from other treatments without organic manure. Li [51] reported that surface mulch could also have effect on soil biological activity, relatively less labeled C with than without mulching, which may have been partly due to the high microbial activity in the mulched treatments [37]. The addition of manure combined with mulching might therefore provide diverse source of nutrients and, result in different types of C sources used by microbes and different effects on microbial functions in general. The microbial utilization of carbohydrates and amino acids was high in FMO soil, whereas the utilization of polymers, phenolic compounds and amines was high in the NMC soil (Fig 5), which may have been due to changes in soil structural properties or the abundant root exudates into the soil by the plants. Increases in microbial activity would improve crop growth and thus a higher demand for nutrient resources by the maize (Fig 7).

Wei et al. [52], however, found that microorganism relative abundance might decrease or not differ significantly with manure-based compost application than those with the same amount of inorganic nitrogen. This finding suggested that the effects of organic farming on soil microbes were complex. Monitoring the long-term effects of mulches and fertilizers on microbial diversity are thus needed in future studies.

### Crop production and nitrogen uptake response to mulching and fertilization

Insufficient precipitation and low soil temperatures often restrict maize growth and development in the dry areas of the western Loess Plateau of China, especially by controlling the rate of development until the meristem emerges from the soil surface. In the present study, film mulching significantly increased maize yield and aboveground biomass by 62.2–70.1%, and gravel mulching increased them by 8.9–14.4% (Fig 7). These results are partly consistent with those of Liu et al. [7], who reported that film mulching promoted maize growth and development, with higher kernel numbers per ear and higher 1000-kernel weight, and with those of Li [51], who demonstrated that gravel–sand mulch improved soil productivity. Mulching may reduce evaporation, reradiation of solar energy and losses of latent and sensible heat [51, 53] and thus increase soil temperature and moisture, promoting earlier germination and plant establishment [24]. The increased interception of radiation would thereby produce more dry matter. The effects of the gravel mulch on grain yield, maize biomass and NUE, however, were not statistically significant compared to the unmulched control (Fig 7), perhaps due to the decrease in the concentrations and amounts of organic matter and other nutrients in the upper soil layer. Luo et al. [38] reported a decrease of 0.51 Mg C hm^-2^ in the organic-C stock under gravel mulching compared with the initial stock after four years. Our study also found relatively minor differences in yield, maize biomass and NUE between the FMO and FMC soils (Fig 7). Different results were reported by Biau et al. [18], but Körschens et al. [54] confirmed our findings. This discrepancy is attributed to the differences in application strategies, soil characteristics or organic resource quality [18, 55]. In the present study, mulching and fertilization had significant effects on nitrogen accumulation prior to silking (*P*<0.05), when 62–72% of the total aboveground nitrogen accumulated (Fig 8). Nitrogen accumulation, however, did not differ significantly between the GMC and NMC soils after silking and at maturity.

### Relationship between microbial properties and crop production

Improved soil hydrothermal conditions can affect microbial and enzymatic activities [48]. Changes in microbial activity may be significantly associated with nutrient availability [56], due to competition for nutrients between plants and microbes [47]. Nieder et al. [57] showed that microbial biomass, as a living part of the soil organic matter, was responsible for nutrient transformation and storage. Allison et al. [58] suggested that enzymatic activity, derived from active microorganisms, could have significant effect on the availability of the most limiting nutrients for meeting microbial metabolic demands, which may stimulate a shift in the composition of microbial communities and accelerate the release of N from manure. This higher MBC observed in FMO soil indicates high inputs and availability of organic matter to soil microorganisms, which would eventually be useful for promoting plant growth. These changes could in turn increase nitrogen uptake by maize, with more inputs of root exudates and other root-borne substances in the mulched compared to unmulched treatments (Fig 8). The significantly higher microbial biomass in the treatment with mulching and manure compared to the unmulched soils was consistent with a higher productivity and nutrient response. Maize yield was thus significantly correlated with MBC, MBN, AWCD and the richness index (*R*^2^ = 0.739**, 0.820**, 0.729** and 0.728**, respectively), indicating that mulching increased plant growth and the temperature of the topsoil [24, 59], which would enhance microbial biomass and the activity of microorganisms and thus lead to higher productivity in areas with limited water availability. In addition, although not measured in this study, it was likely that the input of readily decomposable substrates to the soil would be increased by the combination of mulching with dairy manure application, to the further processing of some nitrogen to the use by the crop [60]. Grain nitrogen accumulation was significantly positively correlated with MBC and MBN (*R*^2^ = 0.721** and 0.803**, respectively), and NUE was positively correlated with MBN (*R*^2^ = 0.630*).

Optimizing soil management in maize production is necessary in arid and semiarid areas where wind erosion is common. Our results have provided baseline information for differences in crop yield that may be partly due to changes in rhizospheric microbial activity with plastic-film mulching plus manure application. Differences in soil microbial and enzymatic activities among systems should be considered when changing farming practices, although monitoring the long-term effects of mulching on soil microbes may be required for a better understanding and management of microbial processes for optimizing crop productivity while maintaining and improving soil quality in this semiarid agroecosystem.

## Conclusion

Different mulching and additional organic application with film mulching resulted in changes in soil microbial activity in the topsoil at the silking stage of maize. Compared with the NMC treatment, the microbial biomass carbon and nitrogen and dehydrogenase activity tended to increase after a long-term GMC, FMC and FMO treatment, whereas the urease activity decreased in the GMC treatment. FMO soil had higher C metabolic activity and shannon diversity index than the NMC, GMC and FMC soils. The FMC and FMO led a greater increase in grain yield, aboveground biomass and nitrogen accumulation than GMC and NMC, while NMC gave the lowest value. Thus, our results indicated that film mulching over gravel mulching might serve as an effective measure for sustainable maize production and optimized nutrient cycling, and plus organic fertilization additions may display improved micro-ecological environments with shifts in microbial biomass and microbial functional diversity in the rainfall-limited semiarid region.

## Supporting Information

S1 Dataset S1 Dataset contains data on precipitation and air temperature, MBC and MBN, dehydrogenase activity and urease activity, AWCD, PCA of CLPP results, relative substrate utilization, the grain yield, biomass and nitrogen use efficiency. (XLS)
